# *N*,*N*′-Diphenyl-1,4-phenylenediamine Antioxidant’s Potential Role in Enhancing the Pancreatic Antioxidant, Immunomodulatory, and Anti-Apoptotic Therapeutic Capabilities of Adipose-Derived Stem Cells in Type I Diabetic Rats

**DOI:** 10.3390/antiox12010058

**Published:** 2022-12-27

**Authors:** Saad Shaaban, Hemdan El-Shamy, Mohamed Gouda, Marwa K. Darwish, Hany M. Abd El-Lateef, Mai M. Khalaf, Ehab I. El-Hallous, Kholoud H. Radwan, Hanan M. Rashwan, Shady G. El-Sawah

**Affiliations:** 1 Department of Chemistry, College of Science, King Faisal University, P.O. Box 380, Al-Ahsa 31982, Saudi Arabia; 2 Department of Chemistry, Organic Chemistry Division, College of Science, Mansoura University, P.O. Box 11432, Mansoura 11001, Egypt; 3 Deanship of Student Affairs, King Faisal University, P.O. Box 380, Al-Ahsa 31982, Saudi Arabia; 4 Department of Psychology, Statistics and Educational Evaluation, Faculty of Education, Al-Azhar University, P.O. Box 11751, Cairo 4434003, Egypt; 5 Chemistry Department (Biochemistry Branch), Faculty of Science, Suez University, P.O. Box 43518, Suez 8151650, Egypt; 6 Department of Medical Laboratories Sciences, College of Applied Medical Sciences, Shaqra University, Al Quwayiyah 19257, Saudi Arabia; 7 Chemistry Department, Faculty of Science, Sohag University, P.O. Box 82524, Sohag 1646130, Egypt; 8 Zoology Department, Faculty of Science, Arish University, El Arish 31111, Egypt; 9 Biology Department, Faculty of Science, Taif University, Taif 21944, Saudi Arabia; 10 Department of Biochemistry, Horus University in Egypt HUE, Damietta 7991164, Egypt

**Keywords:** apoptosis, antioxidants, inflammation, diabetes, stem cells, oxidative stress

## Abstract

Mesenchymal stem cells (MSCs) are considered to be a promising therapeutic protocol for diabetes mellitus (DM) management. The latter is attributed to their differentiation potentiality to pancreatic β-cells, angiogenesis, and immune-modulatory capabilities by releasing various paracrine factors. Interestingly, antioxidant co-administration increased the MSCs’ hypoglycemic and regenerative activities. Thus, this study aims to evaluate the therapeutic implication of type 1 DM after the co-administration of adipose tissue-derived-MSCs (AD-MSCs) and *N*,*N*′-d iphenyl-1,4-phenylenediamine (DPPD), compared to the single injection of either of them alone. In our four week long experiment, six rat groups were used as control, DPPD (250 mg/kg, i.p.), STZ-diabetic (D), D+DPPD, D+AD-MSCs (1 × 10^6^ cell/rat, i.p.), and D+AD-MSCs+DPPD groups. Within this context, a single injection of AD-MSCs or DPPD into diabetic rats showed significant pancreatic anti-inflammatory, immunomodulation, antioxidant, and anti-apoptotic capacities, superior to AD-MSCs injection. However, AD-MSCs and DPPD co-administration into diabetic rats manifested the highest hypoglycemic and pancreatic regenerative activities in managing diabetes compared to the single shot of AD-MSCs or DPPD. These results highlight the synergetic role of DPPD as an antioxidant in enhancing AD-MSCs’ therapeutic applications.

## 1. Introduction

Diabetes Mellitus (DM) is among the deadliest silent diseases and the top ten causes of death in adults, with 4.2 million deaths yearly worldwide [1]. It is a chronic, non-communicable, and debilitating illness associated with diverse complications. It affects all ages and races, leading to reduced life expectancy. Its high mortality puts a massive burden on the medical and socio-technological infrastructure. Furthermore, the worldwide economic burden in 2021 was estimated to be 727 billion USD for DM-related expenditure [2]. Generally, there are two types of DM: type 1 diabetes (T1DM) and type 2 diabetes (T2DM). The former is less common, accounting for ≥10% of diabetic patients. Furthermore, T2DM is more prevalent and accounts for ≤90% of all cases worldwide. The β cells dysfunction is the leading cause of T1DM due to the abnormal interaction between immune cells and pancreatic islets [3]. 

In T1DM patients, transplantation of islet and pancreas have evolved as a prominent strategy because insulin (exogenous) injection cannot mimic the naturally secreted insulin by the normal pancreas. However, these strategies’ widespread application faces many challenges, including transplant complications, shortage of human pancreas and islets donors, limited procedural availability, and high cost [4]. In this context, MSCs are an attractive option for cell-based therapy in regenerative medicine and various inflammatory as well as autoimmune diseases such as DM [5,6]. Furthermore, MSCs studies derived from adipose tissue (AD-MSCs) have gained much attention owing to their easy isolation in large quantities, marked abilities to retain their immunomodulatory characteristics, and high amounts of extracellular matrix components production potentiality [7]. However, despite the AD-MSCs’ considerable success, there is still room for improvement to attain full clinical capacities [8]. 

Many previous clinical and experimental studies highlighted that oxidative stress greatly influences DM development since excessive free radicals’ production coupled with the marked antioxidant defense mechanisms disturbance had led to increased lipid peroxidation, resulting in the progression of various diabetic complications [9]. Interestingly, nonspecific inflammation is accompanied by the production of reactive oxygen species (ROS) at the ischemic injury site, which in turn causes the loss of the transplanted MSCs [10]. Evidence suggests that MSCs are very sensitive to oxidative stress (OS) and ROS. The latter negatively influence the MSCs’ development and impair their self-renewal and differentiation capacity into target cells and direct reprogramming events [8,11,12,13,14]. 

Accordingly, antioxidant co-administration is highly desirable to stimulate MSCs proliferation and counteract ROS implications damaging effect [15]. Furthermore, recent studies on MSCs have shown that antioxidant co-administration supported the overall cell reprogramming-based therapeutic strategies through mitigating OS and enhancing their potency, survival, and differentiation. Thus, opening a new era for the antioxidant’s applications in biomedicine [13,16]. 

**Figure 1:**
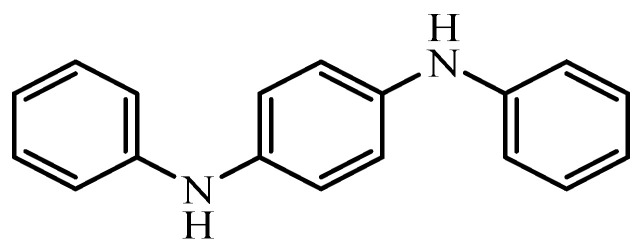
Antioxidants *N*,*N*′-diphenyl-1,4-phenylenediamine.

Within this context, several antioxidants were tested for their potential to maintain stem cells’ viability and differentiation capacities. These included both natural (e.g., l-ascorbic acid-2-phosphate, melatonin, *N*-acetyl-l-cysteine, and resveratrol) and synthetic antioxidants (e.g., Tempol) (Figure 1) [12,13,14,15,16]. Several lines of evidence suggest that melatonin (a natural pineal gland-secreted antioxidant) may be essential in regulating MSCs commitment and differentiation. Melatonin administration was found to protect MSCs from oxidation, inflammation, apoptosis, ischemia, and aging [17,18,19,20]. In another study by Refat et al. [21], a novel (Q/Zn) complex and MSCs co-administration markedly alleviated a series of diabetic complications. In addition, they succeeded in alleviating hyperglycemia and restoring the normal pancreatic structure and function.

Herein, as far as we know, the synthetic antioxidant DPPD (Figure 1) was used for the first time in DM management owing to its promising redox activities. DPPD is phenyl *N*-disubstituted p-phenylenediamine. Its frequently used as an efficient antioxidant in numerous industries (e.g., dyes, rubber, oils, detergents, plastics, and tires) owing to its air and moisture stability. Moreover, DPPD is also used in medicine as an intracellular to improve the microsomal enzymes system efficiency and enhances the pool of lipid-soluble antioxidants. Additionally, it inhibits nephrotoxicity, collagen deposition, lipid peroxidation, and histopathological damage. From a chemistry point of view, the DPPD antioxidant activity arises from its hydrogen donation capacity. The latter disrupts the autocatalytic cycle, thus diminishing ROS production and protecting cells from apoptosis [22,23,24]. Moreover, the DPPD acute oral toxicity is low, i.e., 18,000 mg/kg in mice, and LD_50_ of 2370 mg/kg bw in rats, ensuring its safety [22,23]. Thus, we intend to assess the effect antioxidant of DPPD in enhancing the regenerative capability of MSCs in the diabetic rats’ pancreas. Moreover, we aim to explore the apoptosis minimization of β-cells to restore normal insulin secretion and alleviate hyperglycemia.

## 2. Materials and Methods

### 2.1. Chemicals

DPPD, streptozotocin (STZ), and MSCs media were acquired from Sigma Aldrich Co., Burlington, MA, USA. All kits were purchased from Biodiagnostic Co., Giza, Egypt.

### 2.2. AD-MSCs Isolation and Preparation 

To isolate allograft AD-MSCs from male Wistar rats (6–8 weeks), fresh subcutaneous adipose tissues were lipoaspirate, washed in H_2_NaO_5_P-buffered saline, and digested by collagenase type I solution (0.075%) at 37 °C for three h with shaking, then centrifuged at 1000× *g* for fifteen minutes. Cells were collected, following discarding the supernatant, as a pellet, and the collagenase digestion reaction was stopped by adding Dulbecco’s Modified Eagle Medium (DMEM) with 10% fetal bovine serum (FBS), 2 mM L-glutamine, and 100 U/mL penicillin with 100 μg/mL streptomycin as an antibiotic. The pellet was incubated at 5% CO_2_ (95% humidity) at 37 °C overnight for selecting adherent cells in the DMEM medium and passaged after ≥70% confluence [4]. 

### 2.3. AD-MSCs Characterization 

Finally, before performing the animal study, International Society Cell Therapy’s (ISCT) minimal criteria were followed to confirm the stemness of the cultured cells through flow cytometric analysis of some negative and positive surface markers to ensure the retaining of their phenotype. First, specific rats’ antibodies (CD11b, CD34, CD45, CD73, CD90, and CD105), bought from Sigma Aldrich, were added to the isolated AD-MSCs. Then, the expression level of these molecules on the cell surface was detected using a FACS Caliber flow cytometer (Becton Dickinson, Franklin Lakes, NJ, USA), and FACSDiva Software was used to analyze the results [25]. 

### 2.4. AD-MSCs Counting

10 µL of suspended cells in 1ml media was taken for counting. 2–10 dilution factor was employed in counting cells. A mixture containing 10 µL trypan blue (0.4%, Lonza, Basel, Switzerland) and 10 µL of cells was stirred well. Cells were then counted using a microscope (Olympus CX31, Center Valley, PA, USA) by adding 10 µL of the previously prepared mixture to a hemocytometer (Neubauer) [26].

### 2.5. Animals’ Maintenance and Grouping

Thirty-six male albino Wistar rats (100–120 g), obtained from the laboratory animal facility at the Faculty of Science, Arish University, North Sinai, Egypt, were kept in an animal house (air-conditioned and pathogen-free), with access to water and chow as well as subjection to 12/12 h darkness/daylight. Diabetes was induced using STZ and confirmed through the detection of glucose levels in blood from the vein of the tail three days after induction. Only rats with fasting blood glucose (FBG) above 250 mg/dl were considered diabetic [27]. Animal treatment ethical protocols were obeyed and governed by the Animal Ethics Committee of the Faculty of Science, Suez Canal University, Ismailia, Egypt (REC111/2022). Following acclimatization for two weeks, animals were split into six groups as follows:

1. Control group: received an intraperitoneal (i.p.) sodium citrate (Na_3_C_6_H_5_O_7_) buffer (pH 4.5, single dose) once.
2. DPPD group: one-time injection with DPPD (250 mg/kg, i.p., single dose) daily for four weeks.
3. Diabetic (D) untreated group: one-time injection with STZ (45 mg/kg, i.p., single dose) dissolved in Na_3_C_6_H_5_O_7_ buffer (pH 4.5).
4. Diabetic DPPD treated group: Injected with DPPD (250 mg/kg, i.p., single dose) daily for four weeks, following diabetes induction confirmation.
5. Diabetic AD-MSCs treated group: Injected once with an intravenous (i.v., single dose) of AD-MSCs (1 × 10^6^ cell/rat), following diabetes induction confirmation.
6. Diabetic AD-MSCs+DPPD treated group: Injected once at day one with AD-MSCs (1 × 10^6^ cell/rat, i.v., single dose) following diabetes induction confirmation, then treated with DPPD (250 mg/kg, i.p., single dose) daily for four weeks.

### 2.6. Samples Collection

Rats were slaughtered under anesthesia (pentobarbital) four weeks after MSCs and DPPD treatments. Blood samples were collected by cardiac puncture, and heparin was used as an anticoagulant for HbA1c assessment. Plasma was separated by centrifugation at 1000× *g* for 15 min. Blood sera were carefully separated and stored for subsequent biochemical analysis at −20 °C. Meanwhile, pancreas specimens were harvested, and an appropriate part was weighed and homogenized in cold distilled water forming 10% (*w*/*v*) homogenate, then kept at −20 °C for later biochemical estimations. However, the remnant parts were labeled and held at −80 °C for subsequent flow cytometric analysis.

### 2.7. Biochemical Assays

Bio Vision Company, USA, ELISA kits were used to detect serum insulin and C-peptide levels, while the estimation of serum glucose, glycosylated hemoglobin (HbA1c), and CRP levels was performed using SPINREACT diagnostics kits in Spain. Some pancreatic antioxidants markers (GSH and total antioxidant capacity (TAC) contents, and GST, SOD, CAT, heme-oxygenase 1 (HO-1) activities), in addition to some OS markers (MDA, H_2_O_2_, and AGEs contents, and XO activity), were all assessed using diagnostic kits (Bio Diagnostic Company, Egypt). All experiments were performed according to the supplier’s instructions. Meanwhile, antibodies kits obtained from Sigma Aldrich Company (USA) were used to determine some pancreatic inflammatory markers (TNF-α, TGF-β, CD95^+^, IL-6), immunity markers (CD4^+^, CD8^+^), and apoptotic markers (annexin V, P53, caspase-3, Bcl-2, and G0/G1%), through flow cytometry. 

### 2.8. Preparation and Staining of Pancreatic Cells for Flow Cytometric Analysis

According to the method of Tribukait et al. [28], a fresh pancreatic tissue specimen was washed with isotonic tris EDTA buffer, 3.029 gm of 0.1 M tris (hydroxymethyl aminomethane (cat. No. T-1378, sigma chemical company), 1.022 gm of 0.07 M sodium chloride (ADWIC) and 0.47 gm of 0.005 M EDTA (cat. No. E-6758, sigma). They were dissolved in 250 mL of distilled water and pH was adjusted at 7.5 using 1N HCl, centrifuged for 10 min at 1800 rpm, then aspire supernatant. Sample subjected to hemolysis, if it was contaminated with blood, by filtered tap water for 10 min. Cells are fixed in ice-cold 96–100% ethanol (1 mL for each sample), then stored in refrigerator. Sigma Aldrich Company (USA) kits were used to assess the percentages of some pancreatic inflammatory markers (TNF-α, TGF-β, CD95+, IL-6), immunity markers (CD4+, CD8+), apoptotic markers (annexin V, P53, caspase-3, Bcl-2, and G0/G1 %), in addition to AD-MSCs surface markers (CD11b, CD31, CD44, CD45, CD73, and CD90). The fluorochrome is directly linked to the primary antibody (FITC conjugate). Cell suspension concentration was adjusted (10 cell/mL) with PBS/BSA buffer pH 7.4. Aliquot the cell suspension into test tubes (100 µL each). Add 7µL antibody for each tube, mix well and incubate for 30 min at room temperature. Wash cells with 2ml of PBS/BSA, then centrifuge for 5 min at 4000 rpm and discard the resulting supernatant. Cells resuspended in 0.2 mL of PBS/BSA. Finally, data were acquired by flow cytometry using FACS Caliber flow cytometer (Becton Dickinson, Franklin Lakes, NJ, USA), and the DNA histograms was obtained and analyzed using software (BD FACSDiva Software, Becton Dickinson) [25]. 

### 2.9. Statistical Analysis

ANOVA followed by Post-Hoc Tukey multiple range tests were used to evaluate the obtained data, using Statistical Package for the Social Sciences (SPSS/21 software version) for Windows. All the data were shown as the mean ± standard error (SE), where *n* = 6 and the percent of changes between treated groups were calculated. The significance level was set at *p* ≤ 0.05 for all statistical tests.

## 3. Results

**Figure 2:**
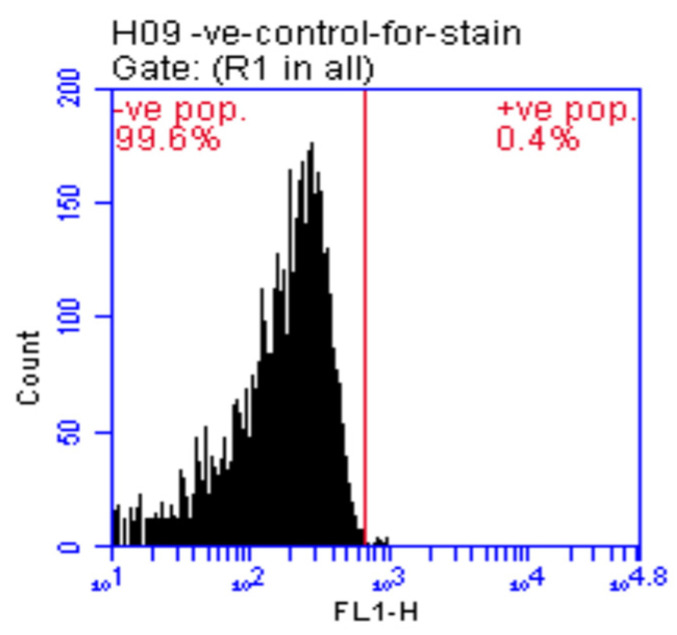

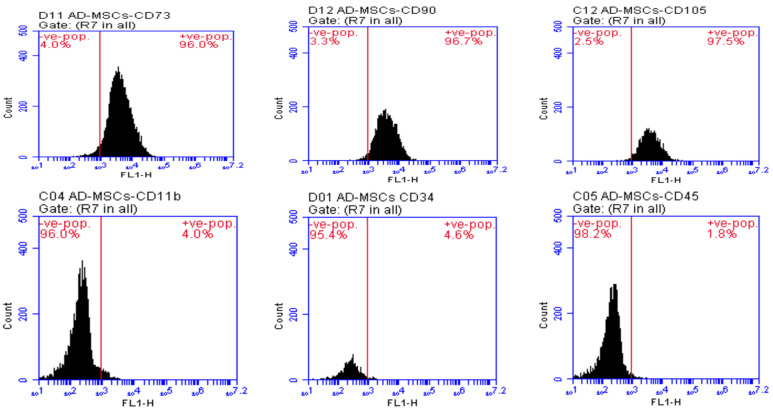
AD-MSCs positive surface markers (CD 73: 96.0%, CD 90: 96.7%, CD 105: 97.5%); AD-MSCs negative surface markers (CD 11b: 4.0%, CD 34: 4.6%, CD 45: 1.8%).

Gates are placed around populations of cells with the relative expression levels of cell specific markers, to investigate, quantify, and identify the negative and positive control cells. Accordingly, cells expressed CD11b, CD45, and CD34 were used as positive control and cells without expressing of CD73, CD90, CD105 were used as negative control. Figure 2 detected very high CD73, CD90, and CD105 expressions that were considered positive, in contrast to deficient CD11b, CD34, and CD45 expressions deemed negative. Such data confirmed the AD-MSCs phenotype identity concomitants with the ISCT minimal criteria. 

**Figure 3:**
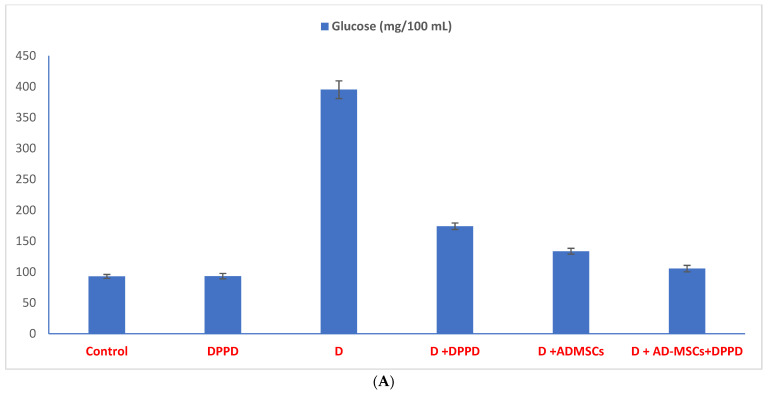

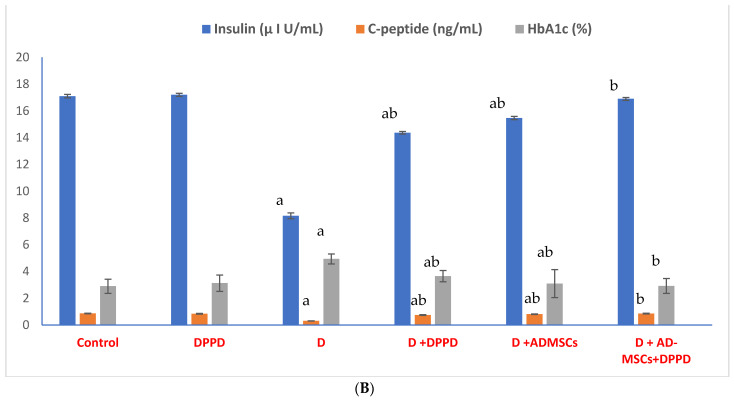
(**A**) Serum glucose levels (mg/100 mL). Values expressed as ±SEM (*n* = 6). ^a, b^ are Significant differences (*p* ≤ 0.05) compared to control, diabetic untreated and DPPD-diabetic treated groups, respectively. % is the percent of change compared to the diabetic group. (**B**) Serum insulin, C-peptide, and HbA1c levels. Values expressed as ±SEM (*n* = 6). ^a, b^ are Significant differences (*p* ≤ 0.05) compared to control, diabetic untreated and DPPD-diabetic treated groups, respectively. % is the percent of change compared to the diabetic group.

Furthermore, Figure 3A,B and Table S1 (Supplementary Information) showed that the diabetic group exhibited significant serum glucose and HbA1c levels increase while necessary insulin and C-peptide declined, compared to the control group. However, all three diabetic treated groups revealed a noticeable enhancement in all measured parameters relative to diabetics. Interestingly, the percentage of change showed that diabetic rats treated with the AD-MSCs+DPPD combination detected the most marked hypoglycemic superiority compared to other treated groups. 

**Figure 4:**
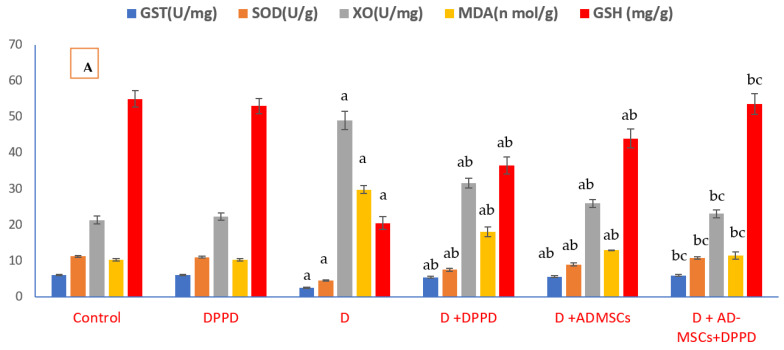

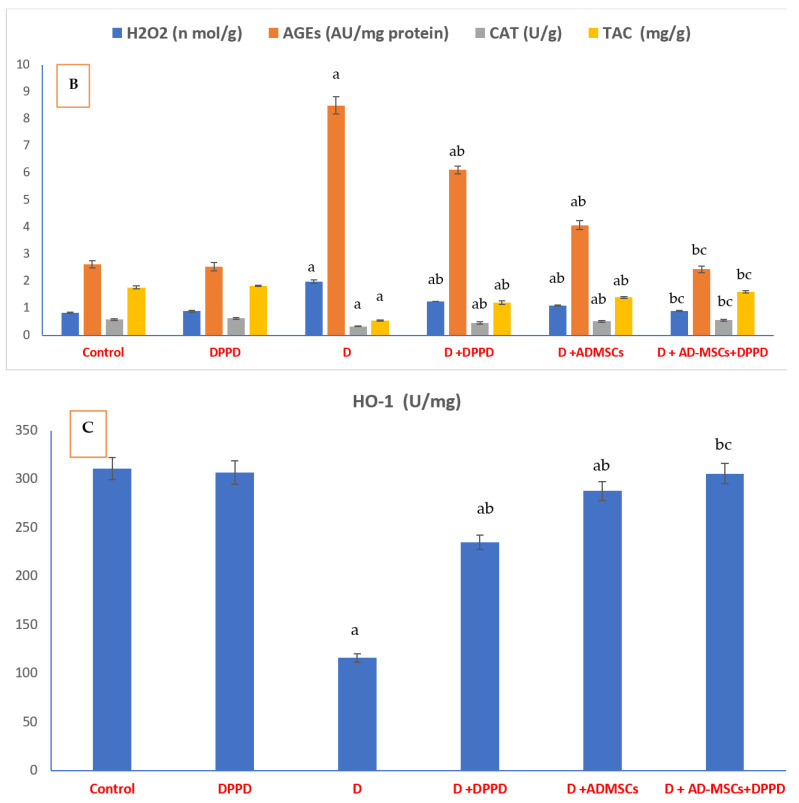
(**A**). Pancreatic oxidative stress (MDA and XO) levels and antioxidant markers (SOD and GST) levels. Values expressed as ±SEM (*n* = 6). ^a, b^ & ^c^ are Significant differences (*p* ≤ 0.05) compared to control, diabetic untreated and DPPD-diabetic treated groups, respectively. % is the percent of change compared to the diabetic group. (**B**). Pancreatic oxidative stress (H_2_O_2_ and AGEs) levels and antioxidant markers (CAT and TAC) levels. Values expressed as ±SEM (*n* = 6). ^a, b^ & ^c^ are Significant differences (*p* ≤ 0.05) compared to control, diabetic untreated and DPPD-diabetic treated groups, respectively. % is the percent of change compared to the diabetic group. (**C**). Pancreatic heme-oxygenase 1 oxidative stress levels. Values expressed as ±SEM (*n* = 6). ^a, b^ & ^c^ are Significant differences (*p* ≤ 0.05) compared to control, diabetic untreated and DPPD-diabetic treated groups, respectively. % is the percent of change compared to the diabetic group.

Figure 4A–C and Table S2 (Supplementary Information) highlighted marked elevations in pancreatic OS markers (MDA, H_2_O_2_, AGEs, and XO) levels and a significant down-regulation in all antioxidant markers (GSH, SOD, CAT, GST, TAC, and HO-1) levels in the diabetic group when compared to the control rats. Additionally, diabetic rats treated with either DPPD or AD-MSCs or both combinations reflected significant oxidative markers decrease. In contrast, critical antioxidant markers increase in the diabetic group. Results of diabetic rats treated with AD-MSCs+DPPD recorded a tremendous antioxidant potentiality to other treatments. 

**Figure 5:**
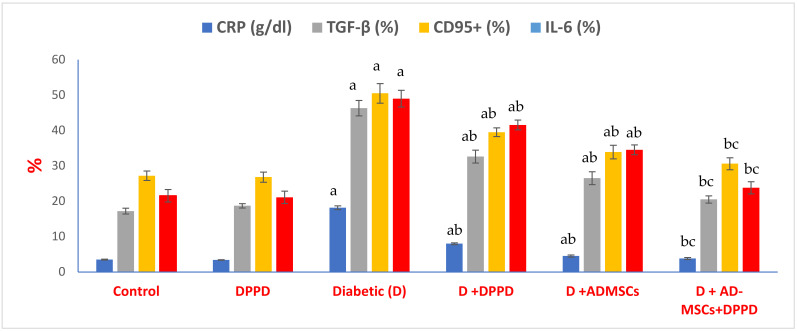
Serum CRP & pancreatic TGF-β, CD95, and IL-6 levels. Values expressed as ±SEM (*n* = 6). ^a, b^ & ^c^ are Significant differences (*p* ≤ 0.05) compared to control, diabetic untreated and DPPD-diabetic treated groups, respectively. % is the percent of change compared to the diabetic group.

Figure 5 and Figure S1a–c and Table S3 (Supplementary Information) illustrate marked rises in pancreatic inflammatory markers (CRP, TGF-β, CD95^+^, and IL-6) %, compared to control. However, these inflammatory markers % exhibited significant declines in all diabetic rats treated groups compared to the diabetic untreated group. Of note, the AD-MSCs+DPPD combination revealed the highest anti-inflammatory efficacy than DPPD or AD-MSCs treatment alone in diabetic rats. 

**Figure 6:**
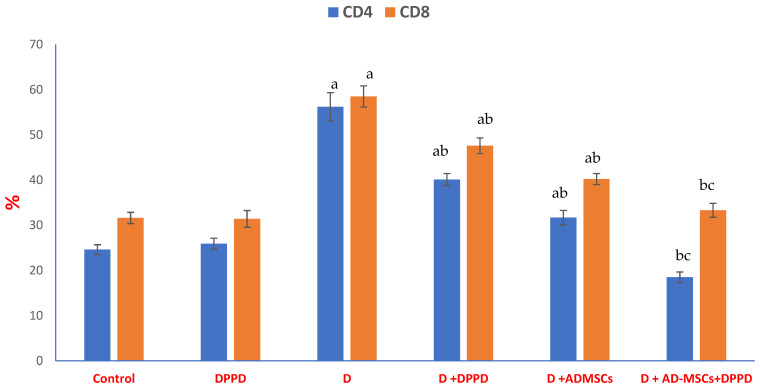
Pancreatic T-Helper (CD 4) and T-Cytotoxic (CD 8) %. Values expressed as ±SEM (*n* = 6). ^a, b^ & ^c^ are Significant differences (*p* ≤ 0.05) compared to control, diabetic untreated and DPPD-diabetic treated groups, respectively. % is the percent of change compared to the diabetic group.

Figure 6 and Figure S2a,b and Table S4 (Supplementary Information) summarized data indicating a considerable elevation in pancreatic immune parameters % in the diabetic rats; compared to normal control rats. Furthermore, relative to the diabetic untreated group, the three diabetic treated groups’ results reported a notable boost in both examined parameters. Importantly, diabetic rats injected with AD-MSCs+DPPD showed the most noticeable enhancement in the pancreatic immune condition compared to a single injection with DPPD or AD-MSCs.

**Figure 7:**
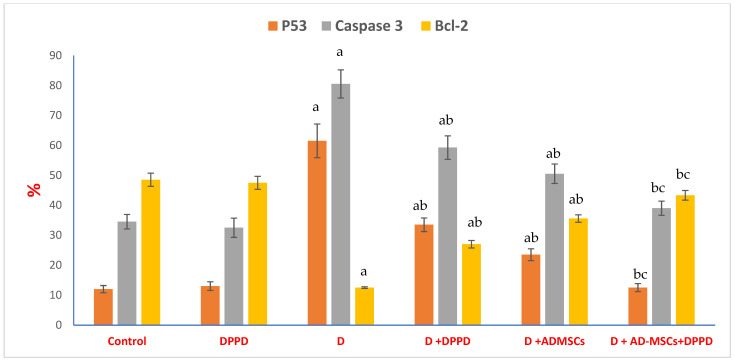
Pancreatic P53, caspase 3, & Bcl-2%. Values expressed as ±SEM (*n* = 6). ^a, b^ & ^c^ are Significant differences (*p* ≤ 0.05) compared to control, diabetic untreated and DPPD-diabetic treated groups, respectively. % is the percent of change compared to the diabetic group.

Table 1 and Table 2, Figure 7, Figures S3a–e, S4 and S5 and Table S5 (Supplementary Information) revealed significant increases in many apoptotic markers (annexin V, P53, and caspase 3%), suggesting a substantial development in the pancreatic cells’ apoptotic status of diabetic rats, associated with a remarkable diminishing in both anti-apoptotic marker (Bcl-2%) and viable pancreatic cells count (G0/G1%); when compared to control. On the contrary, all diabetic treated group data reflected a significant improvement in all tested indicators compared to the diabetic untreated group. Notably, the AD-MSCs+DPPD combination treatment revealed the most potent pancreatic anti-apoptotic excellency in diabetic rats, compared to either DPPD or AD-MSCs treatments alone.

## 4. Discussion

Since the primary diabetes induction mechanism depends on increasing the OS status in the pancreas, we suggest the mechanism of treatment was somehow complicated and connected with the results; since Co-administration of DPPD with AD-MSCs significantly increased the pancreatic antioxidants contents which resulted directly in the significant suppression of the OS status in the pancreas of diabetic rats. These events might, in turn, decrease the inflammation and adjust the immunity of the diabetic pancreas resulting in a significant increase in several viable pancreatic cells with suppression of pancreatic apoptosis. Taken together, it was suggested these changes enhance the hyperglycemic status and control the increased blood glucose level. Interestingly, MSCs transplantation was found to be capable of controlling blood glucose in addition to decreasing the daily insulin requirement in diabetic animals via normalizing both serum HbA1C and C-peptide levels, which could be attributed to the trans-differentiation potentiality of the transplanted MSCs into insulin-producing cells (IPCs) [8,29]. However, Abdel Fattah et al. [30] and Aminzadeh et al. [31] suggested that the MSCs’ hypoglycemic ability and their notable antioxidant activity in suppressing OS status is because of the persistent hyperglycemia, shown by the increased TAC, HO-1, GSH-Px-1, 3 and 4, SOD-1 and 3, CAT, and GSH mRNA expression levels coupled with the decreased ROS and MDA levels, compared to diabetic untreated rats. Theoretically, MSCs treatment can manage hyperglycemia in TIDM by restoring and raising the β-cell mass via the following steps (1) increasing β-cell proliferation and protection accompanied by decreasing their apoptosis; (2) modification of the local microenvironment through the release of various chemokines and cytokines capable in inducing the regeneration of endogenous β-cells; (3) modulation of β-cells’ autoimmunity [32].

Regarding the first step, according to Wang et al. [33], the increased β-cell mass following AD-MSC injection is accompanied by less inflammation in the pancreas, confirmed via the declined expression of TNF-α. Additionally, AD-MSCs could induce both β-islets count and function restoration by minimizing their apoptotic rate by lowering the activity of the pro-apoptotic caspase-3 enzyme. In addition, islets vascularization can be promoted through the increased production of some paracrine angiogenic factors (VEGF, IGF-1, and HGF) that could participate in the β-islets regeneration [34]. In line with the second step, it has been found that MSCs injection can release many growth factors, immunosuppressive molecules, and inflammatory cytokines, such as nitric oxide (NO), IL-10, TGF-β, IL-6, TNF-α, and HO-1; capable in modulating and improving the surrounding microenvironment, which could consequently induce the pancreatic tissue repair process [8,29]. Thus, since MSCs’ immunomodulatory potential depends on both the cytokine and inflammation status, it was supposedly plastic [17,35]. 

In concomitant to the third step, MSCs were proved to have a unique potential to modulate and/or suppress the autoimmune responses in T1DM. Many reports have suggested the MSCs’ immunotolerance capabilities’ underlying mechanism might be due to their (i) hypo-immunogenicity, (ii) microenvironment immune suppression [36], (iii) inducing macrophages conversion ability from pro-inflammatory type 1 to anti-inflammatory phenotype [35,37], (iv) ability to minimize the incompatible lymphocytes alloreactivity, (v) T-cell modulation capability and finally (vi) mixed lymphocyte reaction suppression through inducing the expansion of T regulatory cells (T-regs) ratio versus other T cells subtypes; via inducing the T-regs generation with simultaneous inhibition of the T helper 1 (Th1) and Th17 cells proliferation [33,38]. 

However, it was proved that a hyperglycemic environment could minimize the MSCs’ proliferative capability by increasing either their senescence or apoptosis [39]. Furthermore, despite certain ROS physiological levels being necessary to maintain the MSCs’ self-renewal potentiality, the increased expression could result in the initiation of significant mitochondrial DNA damage, chromosomal aberrations, and impaired MSCs trans-differentiation. Thus, MSCs-antioxidant co-administration was suggested not only to mitigate OS and improve stem cell survival but also to support these cells’ reprogramming, differentiation, and potency [16]. It should be noted, however, that the actions of such antioxidants may be not only through their ROS-inhibiting abilities but also due to independent mechanisms of the classical antioxidant pathways [13]. In agreement with our results, Nabil et al. [24] reported DPPD ameliorates the HgCl_2_-induced OS, inflammation, and auto-immunity in both liver and kidney in rats, shown through the declined MDA level, significant antioxidant (CAT, GSH, and SOD) increased expressions, reversed TGF-*β*, CD4^+^, and CD8^+^ percent alterations toward the normal ranges. Similar DPPD results were confirmed previously by Kawai et al. [22], Omar et al. [23,40], and Zahran et al. [26,41]. Such results were similar and supported to the previous studies regarding diabetes treatment with MSCs and antioxidants co-administration. According to Kadry et al. [42], the administration of either MSCs alone or in combination with melatonin to diabetic rats resulted in a marked body and pancreas weight increase, in addition to significant glucose, insulin, MDA levels, and TAC improvement. Results also reported an apparent pancreatic inflammation and apoptosis reduction, as shown by substantial progress in TNF-α, IL-10, and caspase-3 levels, resulting in a reduced number of damaged beta cells. However, a melatonin and stem cell co-treatment was more effective than stem cells alone, which could be attributed to the melatonin antioxidant potentiality in maximizing both MSCs’ vitality and efficiency. In similar studies, AD-MSCs preconditioned with resveratrol was found to enhance the AD-MSCs viability in diabetic rats and to maximize their antifibrotic action in diabetic cardiomyopathy [43], liver dysfunction [44], and damaged pancreas [45]. On the other hand, MSCs in combination with quercetin therapy revealed a potent synergistic effect against STZ-induced hyperglycemia, oxidative stress, and genotoxicity in the pancreas and lungs of diabetic rats, which could be considered to be a potential ameliorative therapy against DM and its complications [21]. 

## 5. Conclusions

Although injection of either AD-MSCs or DPPD antioxidant alone into type 1 diabetic rats resulted clearly in reducing the elevated blood glucose level with a marked superiority for AD-MSCs injection, AD-MSCs+DPPD combination treatment revealed the most potent hypoglycemic activity with the greatest pancreatic antioxidant, anti-inflammatory, immunomodulatory, anti-apoptotic and regenerative excellencies in diabetic rats. Thus, such results encourage this combined treatment to alleviate other diabetic tissue complications.

## Figures and Tables

**Table 1:** Pancreatic cell cycle (G0/G1)%.

		Control	DPPD	Diabetic (D)	D+DPPD	D+AD-MSCs	D+AD- MSCs+DPPD
G0/G1 phase (%)	Mean ± SEM	91.70 ± 3.16	92.30 ± 3.37	57.50 ± 1.22 ^**a**^	75.50 ± 2.43 ^**ab**^	85.00 ± 2.63 ^**ab**^	93.00 ± 3.25 ^**bc**^
S Phase (%)	Mean ± SEM	2.50 ± 0.01	2.70 ± 0.02	11.00 ± 0.03 ^**a**^	6.50 ± 0.02 ^**ab**^	1.00 ± 0.01 ^**ab**^	2.50± 0.03 ^**bc**^
G2/M phase (%)	Mean ± SEM	2.80 ± 0.02	2.00 ± 0.02	6.50 ± 0.01 ^**a**^	1.70 ± 0.03 ^**ab**^	3.00 ± 0.01 ^**ab**^	2.70 ± 0.02 ^**bc**^

Values expressed as ±SEM (*n* = 6). **^a, b & c^** are Significant differences (*p* ≤ 0.05) compared to control, diabetic untreated and DPPD-diabetic treated groups, respectively. % is the percent of change compared to the diabetic group.

**Table 2:** Pancreatic annexin V%.

		Control	DPPD	Diabetic (D)	D+DPPD	D+AD-MSCs	D+AD- MSCs+DPPD
Viable cells (%)	Mean ± SEM	93.70 ± 3.16	92.50 ± 3.37	33.20 ± 1.22 ^**a**^	63.50 ± 2.43 ^**ab**^	72.90 ± 2.63 ^**ab**^	93.00 ± 2.25 ^**bc**^
Early apoptosis (%)	Mean ± SEM	0.50 ± 0.21	1.40 ± 0.45	3.50 ± 0.63 ^**a**^	15.00 ± 0.26 ^**ab**^	13.90 ± 0.98 ^**ab**^	0.60 ± 0.33 ^**bc**^
Late apoptosis (%)	Mean ± SEM	4.60 ± 1.42	4.80 ± 1.22	29.00 ± 2.71 ^**a**^	21.80 ± 2.93 ^**ab**^	12.50 ± 2.26 ^**ab**^	6.50 ± 1.37 ^**bc**^
Necrosis (%)	Mean ± SEM	1.50 ± 0.12	1.40 ± 0.16	35.70 ± 2.21 ^**a**^	0.20 ± 0.02 ^**ab**^	0.10 ± 0.01 ^**ab**^	0.30 ± 0.01 ^**bc**^

Values expressed as ±SEM (*n* = 6). **^a, b & c^** are Significant differences (*p* ≤ 0.05) compared to control, diabetic untreated and DPPD-diabetic treated groups, respectively. % is the percent of change compared to the diabetic group.

## Data Availability

Data is contained within the article and the supplementary materials.

## Supplementary Materials

The following supporting information can be downloaded at: https://www.mdpi.com/article/10.3390/antiox12010058/s1, Figure S1: (a–c). Pancreatic TGF-β, CD95, and IL-6 levels; Figure S2: (a,b). Pancreatic T-Helper (CD 4^+^) and T-Cytotoxic (CD 8^+^) %.; Figure S3: (a–e). Pancreatic annexin V, P53, caspase 3, Bcl-2 & viable cells (G0/G1) %; Figure S4: Pancreatic cell cycle %. Values expressed as ±SEM (n = 6). ^a, b & c^ are Significant differences (*p* ≤ 0.05) compared to control, diabetic untreated and DPPD-diabetic treated groups, respectively. % is the percent of change compared to the diabetic group; Figure S5: Pancreatic annexin V%. Values expressed as ±SEM (n = 6). ^a, b^ & ^c^ are Significant differences (*p* ≤ 0.05) compared to control, diabetic untreated and DPPD-diabetic treated groups, respectively. % is the percent of change compared to the diabetic group; Table S1: Serum glucose, insulin, C-peptide, and HbA1c levels. Table S2: Pancreatic OS and antioxidant markers levels. Table S3: Serum CRP, TGF-β, CD95, and IL-6 levels. Table S4: Pancreatic T-Helper (CD 4^+^) and T-Cytotoxic (CD 8^+^) %. Table S5: Pancreatic P53, caspase 3, & Bcl-2%.
