# Supplementary material for: N,N′-Diphenyl-1,4-phenylenediamine Antioxidant’s Potential Role in Enhancing the Pancreatic Antioxidant, Immunomodulatory, and Anti-Apoptotic Therapeutic Capabilities of Adipose-Derived Stem Cells in Type I Diabetic Rats
Source: Antioxidants (Basel). 2022 Dec 27;12(1):58. doi: 10.3390/antiox12010058 (PMC9854452; doi:10.3390/antiox12010058)
Supplement: Supplementary file 1 [file antioxidants-12-00058-s001.zip › antioxidants-2001960-supplementary.pdf]

N,*N′*-Diphenyl-1,4-phenylenediamine Antioxidant's Potential Role in Enhancing the Pancreatic Antioxidant, Immunomodulatory, and Anti-Apoptotic Therapeutic Capabilities of Adipose-Derived Stem Cells in Type I Diabetic Rats

Saad Shaaban [1,2,*], Hemdan El-Shamy [3,4], Mohamed Gouda [1], Marwa K. Darwish [5,6], Hany M. Abd El-Lateef [1,7], Mai M. Khalaf [1,7], Ehab I. El-Hallous [8,9], Kholoud H. Radwan [10], Hanan M. Rashwan [8] and Shady G. El-Sawah [8,*]

[1] Department of Chemistry, College of Science, King Faisal University, P.O. Box 380, Al-Ahsa 31982, Saudi Arabia
[2] Department of Chemistry, Organic Chemistry Division, College of Science, Mansoura University, P.O. Box 11432, Mansoura 11001, Egypt
[3] Deanship of Student Affairs, King Faisal University, P.O. Box 380, Al-Ahsa 31982, Saudi Arabia
[4] Department of Psychology, Statistics and Educational Evaluation, Faculty of Education, Al-Azhar University, P.O. Box 11751, Cairo 4434003, Egypt
[5] Chemistry Department (Biochemistry Branch), Faculty of Science, Suez University, P.O. Box 43518, Suez 8151650, Egypt
[6] Department of Medical Laboratories Sciences, College of Applied Medical Sciences, Shaqra University, Al Quwayiyah 19257, Saudi Arabia
[7] Chemistry Department, Faculty of Science, Sohag University, P.O. Box 82524, Sohag 1646130, Egypt
[8] Zoology Department, Faculty of Science, Arish University, North Sinai 31111, Egypt
[9] Biology Department, Faculty of Science, Taif University, Taif 21944, Saudi Arabia
[10] Department of Biochemistry, Horus University in Egypt HUE, Damietta 7991164, Egypt
* Correspondence: sibrahim@kfu.edu.sa or dr_saad_chem@mans.edu.eg (S.S.); dr.shadygamal83@gmail.com (S.G.E.-S.)

Table S1. Serum glucose, insulin, C-peptide, and HbA1c levels.

		Control	DPPD	Diabetic (D)	D + DPPD	D + ADMSCs	D + AD-MSCs+DPPD
**Glucose (mg/ 100 ml)**	**Mean**	92.83	93.33	395.20	174.20	133.66	105.50
	**±SEM**	± 3.14	± 4.28	±14.33 [a]	±5.24 [ab]	±4.65 [ab]	±5.17 [bc]
	**%**				-55.92	-66.17	-73.30
**Insulin (µ I U/ml)**	**Mean**	17.10	17.20	8.16	14.37	15.47	16.90
	**±SEM**	± 0.53	± 0.61	±0.37 [a]	±0.42 [ab]	± 1.05 [ab]	±0.56 [bc]
	**%**				+76.10	+89.58	+107.10
**C-peptide (ng/ml)**	**Mean**	0.87	0.84	0.31	0.75	0.81	0.86
	**±SEM**	± 0.02	± 0.03	± 0.01 [a]	± 0.03 [ab]	± 0.02 [ab]	± 0.03 [bc]
	**%**				+141.93	+161.29	+177.41
**HbA1c (%)**	**Mean**	2.90	3.13	4.94	3.65	3.10	2.92
	**±SEM**	± 0.13	± 0.12	± 0.22 [a]	± 0.10 [ab]	± 0.12 [ab]	± 0.11 [bc]
	**%**				- 26.11	- 37.24	- 40.89

Values expressed as ± SEM (n = 6). [a,b & c] are Significant differences ($P \leq 0.05$) compared to control, diabetic untreated and DPPD-diabetic treated groups, respectively. % is the percent of change compared to the diabetic group.

Table S2. Pancreatic OS and antioxidant markers levels.

		Control	DPPD	Diabetic (D)	D + DPPD	D + AD-MSCs	D + AD-MSCs+DPPD
**MDA (n mol/g)**	**Mean ±SEM**	10.30 ± 0.32	10.36 ± 0.28	29.84 ± 1.16	18.08 ±1.32 [ab]	13.00 ± 0.08 [ab]	11.50 ± 1.05 [bc]
	**%**				-39.41	-56.43	-61.46
**H_2O_2 (mM/g)**	**Mean ±SEM**	0.83 ± 0.02	0.89 ± 0.03	1.98 ± 0.06 [a]	1.25 ±0.02 [ab]	1.10 ± 0.02 [ab]	0.90 ± 0.02 [bc]
	**%**				-36.86	-44.44	-54.54
**AGEs (AU/mg protein)**	**Mean ±SEM**	2.62 ± 0.14	2.53 ±0.15	8.50 ± 0.33 [a]	6.11 ± 0.14 [ab]	4.07 ± 0.17 [ab]	2.44 ± 0.12 [bc]
	**%**				-28.11	-52.11	-71.29
**XO (U/mg)**	**Mean ±SEM**	21.40 ± 1.04	22.33 ± 1.02	49.00 ± 2.52 [a]	31.66 ±1.39 [ab]	26.00 ± 1.13 [ab]	23.13 ± 1.08 [bc]
	**%**				-35.38	-46.93	-52.79
**GSH (mg/g)**	**Mean ±SEM**	55.00 ± 2.24	53.00 ± 2.13	20.56 ± 1.82 [a]	36.50 ±2.36 [ab]	44.00 ± 2.64 [ab]	53.60 ± 2.93 [bc]
	**%**				+77.52	+114.00	+160.70
**SOD (U/g)**	**Mean ±SEM**	11.33 ± 0.25	11.10 ± 0.22	4.56 ± 0.17 [a]	7.60 ±0.43 [ab]	9.08 ± 0.43 [ab]	10.80 ± 0.32 [bc]
	**%**				+66.66	+99.12	+136.84
**CAT (U/g)**	**Mean ±SEM**	0.58 ± 0.03	0.62 ± 0.04	0.33 ± 0.01 [a]	0.46 ±0.04 [ab]	0.51 ± 0.03 [ab]	0.56 ± 0.03 [bc]
	**%**				+39.39	+54.54	+69.69
**GST (U/mg)**	**Mean ±SEM**	6.15 ± 0.11	6.13 ± 0.13	2.60 ± 0.12 [a]	5.50 ±0.29 [ab]	5.70 ± 0.22 [ab]	6.02 ± 0.21 [bc]
	**%**				+111.53	+119.23	+131.53
**TAC (mg/g)**	**Mean ±SEM**	1.77 ± 0.06	1.83 ± 0.02	0.55 ± 0.02 [a]	1.20 ±0.07 [ab]	1.40 ± 0.03 [ab]	1.60 ± 0.05 [bc]
	**%**				+118.18	+154.54	+190.90
**HO-1 (U/mg)**	**Mean ±SEM**	311.00 ± 11.34	307.00 ±12.38	116.00 ± 4.28 [a]	235.00 ±7.39 [ab]	288.00 ± 9.76 [ab]	306.00 ± 10.12 [bc]
	**%**				+102.58	+148.27	+163.79

Values expressed as ± SEM (n = 6). [a,b & c] are Significant differences ($P \le 0.05$) compared to control, diabetic untreated and DPPD-diabetic treated groups, respectively. % is the percent of change compared to the diabetic group.

Table S3. Serum CRP, TGF-β, CD95, and IL-6 levels.

		Control	DPPD	Diabetic (D)	D + DPPD	D + ADMSCs	D + AD-MSCs+DPPD
**CRP (g/dl)**	**Mean**	3.50	3.40	18.16	8.00	4.50	3.80
	**±SEM**	± 0.11	± 0.09	± 0.54 [a]	± 0.26 [ab]	± 0.32 [ab]	± 0.28 [bc]
	**%**				-55.94	-75.22	-79.07
**TGF-β (%)**	**Mean**	17.00	18.50	46.30	32.60	26.50	20.50
	**±SEM**	± 0.82	± 0.63	± 2.17 [a]	± 1.82 [ab]	± 1.82 [ab]	± 1.03 [bc]
	**%**				-29.58	-42.76	-55.72
**$CD95^+$ (%)**	**Mean**	27.20	26.80	50.50	39.50	33.90	30.60
	**±SEM**	± 1.34	± 1.45	± 2.77 [a]	± 1.22 [ab]	± 1.93 [ab]	± 1.68 [bc]
	**%**				-21.78	-32.87	-39.40
**IL-6 (%)**	**Mean**	21.55	21.10	49.00	41.55	34.55	23.80
	**±SEM**	± 1.76	± 1.76	± 2.34 [a]	± 1.38 [ab]	± 1.38 [ab]	± 1.72 [bc]
	**%**				-15.20	-29.48	-51.42

Values expressed as ± SEM (n = 6). [a,b & c] are Significant differences ($P \leq 0.05$) compared to control, diabetic untreated and DPPD-diabetic treated groups, respectively. % is the percent of change compared to the diabetic group.

Table S4. Pancreatic T-Helper (CD 4^{+}) and T-Cytotoxic (CD 8^{+}) %.

		Control	DPPD	Diabetic (D)	D + DPPD	D + AD-MSC	D + AD-MSCs+DPPD
**$CD4^{+}$ (%)**	**Mean**	24.60	25.90	56.20	40.10	31.70	18.50
	**±SEM**	± 1.10	±1.22	± 3.15 [a]	±1.32 [ab]	± 1.58 [ab]	± 1.16 [bc]
	**%**				-28.64	-43.59	-67.08
**$CD8^{+}$ (%)**	**Mean**	31.60	31.40	58.50	47.60	40.20	33.30
	**±SEM**	± 1.25	±1.85	± 2.35 [a]	±1.74 [ab]	± 1.22 [ab]	± 1.54 [bc]
	**%**				-18.63	-31.28	-43.07

Values expressed as ± SEM (n = 6). [a,b & c] are Significant differences ($P \leq 0.05$) compared to control, diabetic untreated and DPPD-diabetic treated groups, respectively. % is the percent of change compared to the diabetic group.

Table S5. Pancreatic P53, caspase 3, & Bcl-2 %.

		Control	DPPD	Diabetic (D)	D + DPPD	D + AD-MSCs	D + AD-MSCs+DPPD
**P53 (%)**	**Mean**	12.00	13.00	61.50	33.50	23.50	12.50
	**±SEM**	± 1.21	± 1.45	± 5.63 [a]	± 2.26 [ab]	± 1.98 [ab]	± 1.33 [b]
	**%**				-45.52	-99.16	-79.67
**Caspase 3 (%)**	**Mean**	34.50	32.50 ± 3.22	80.50	59.25	50.50	39.00
	**±SEM**	± 2.42		± 4.71 [a]	± 3.93 [ab]	± 3.26 [ab]	± 2.37 [b]
	**%**				-26.39	-37.26	-51.55
**Bcl-2 (%)**	**Mean**	48.50	47.50 ± 2.16	12.50	27.00	35.55	43.30
	**±SEM**	± 2.19		± 0.27 [a]	±1.24 [ab]	± 1.27 [ab]	± 1.62 [b]
	**%**				+116.00	+184.40	+246.40

Values expressed as ± SEM (n = 6). [a,b & c] are Significant differences ($P \leq 0.05$) compared to control, diabetic untreated and DPPD-diabetic treated groups, respectively. % is the percent of change compared to the diabetic group.

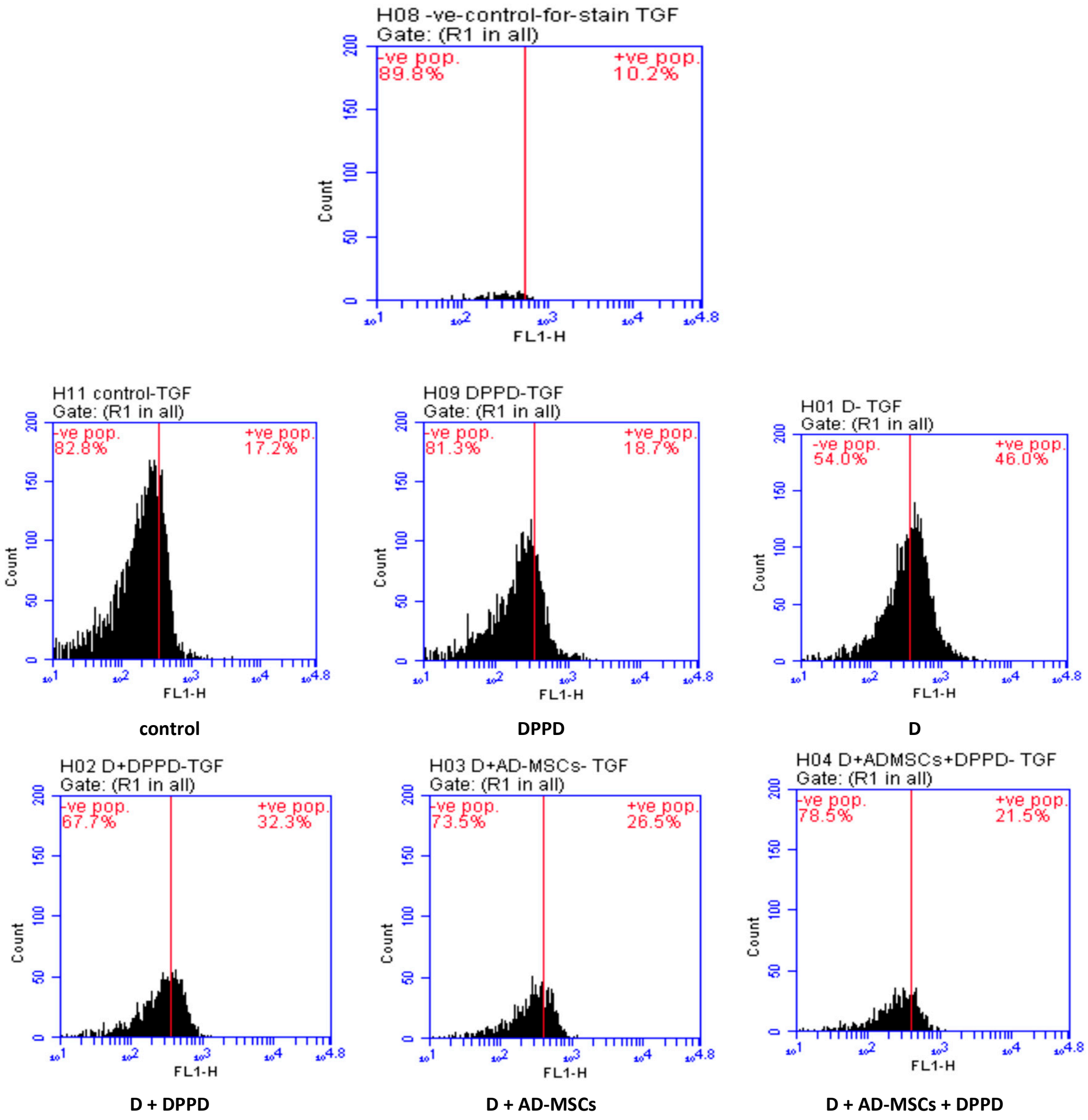

Figure S1a. Pancreatic TGF-β %

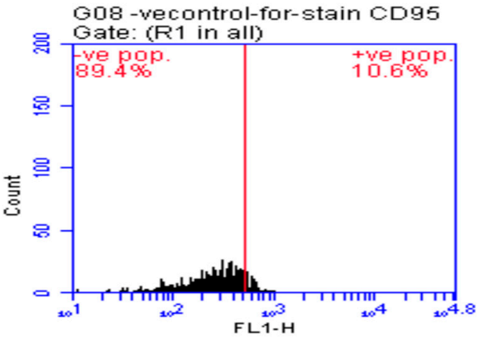

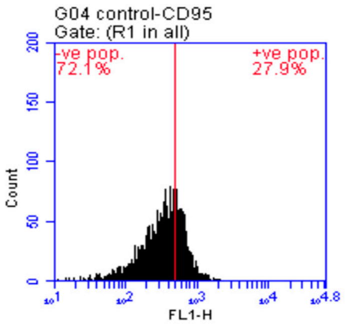

control

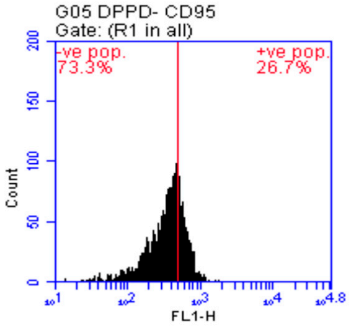

DPPD

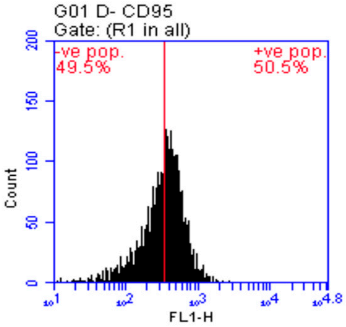

D

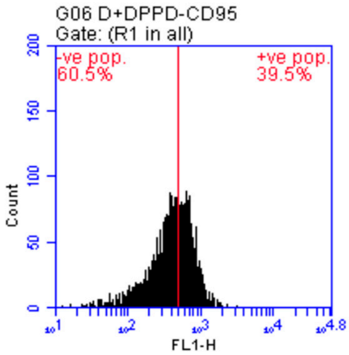

D + DPPD

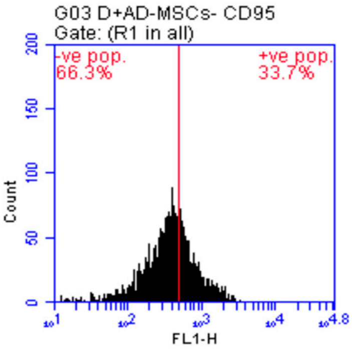

D + AD-MSCs

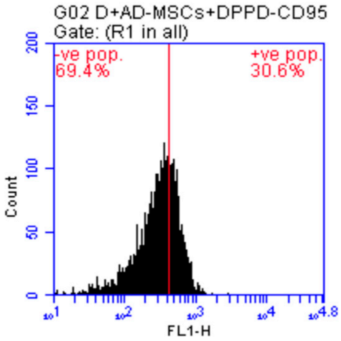

D + AD-MSCs + DPPD

Figure S1b. Pancreatic CD95+ %.

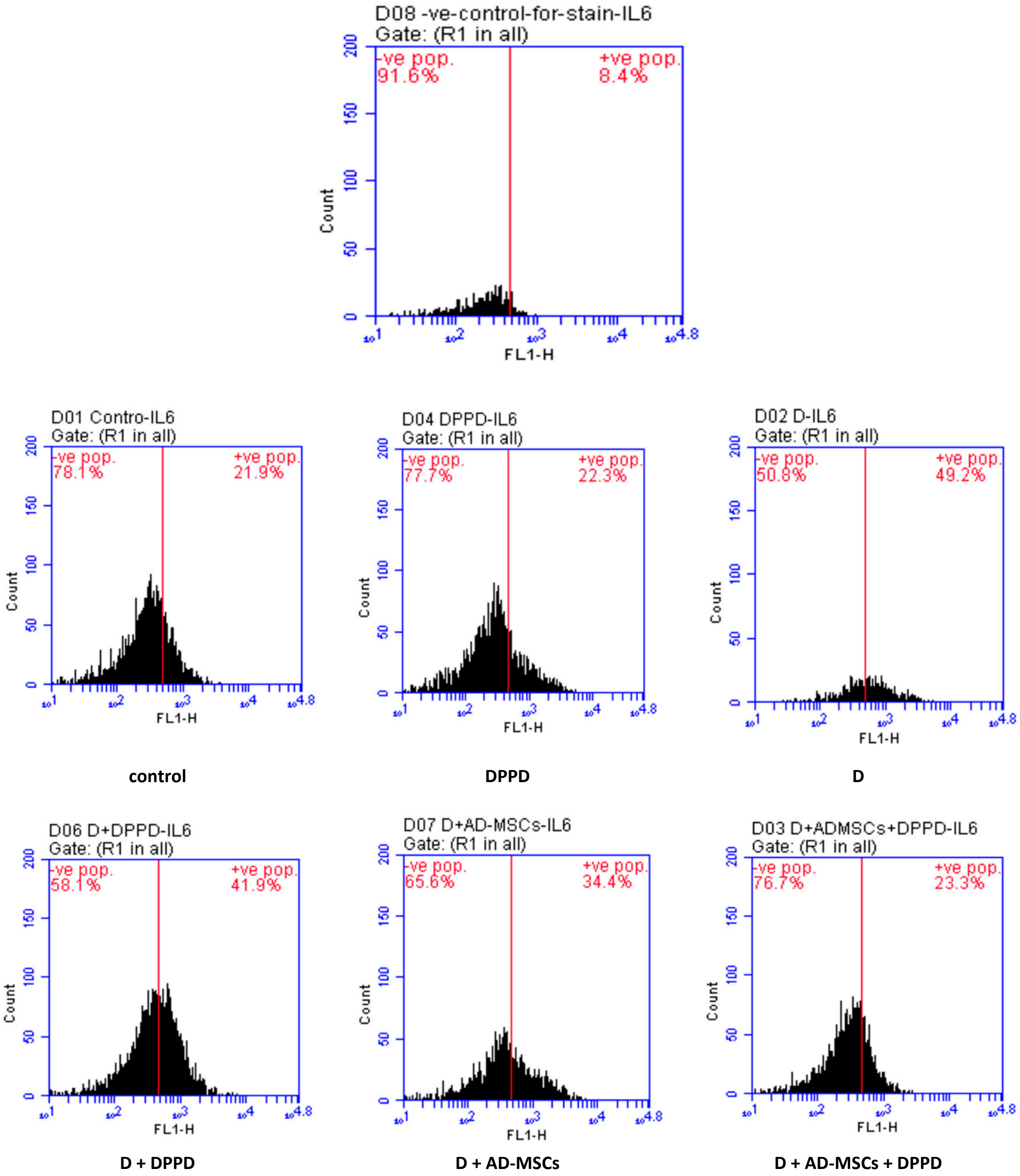

Figure S1c. Pancreatic IL-6 %

Figure S1 (a-c). Pancreatic TGF-β, CD95, and IL-6 levels.

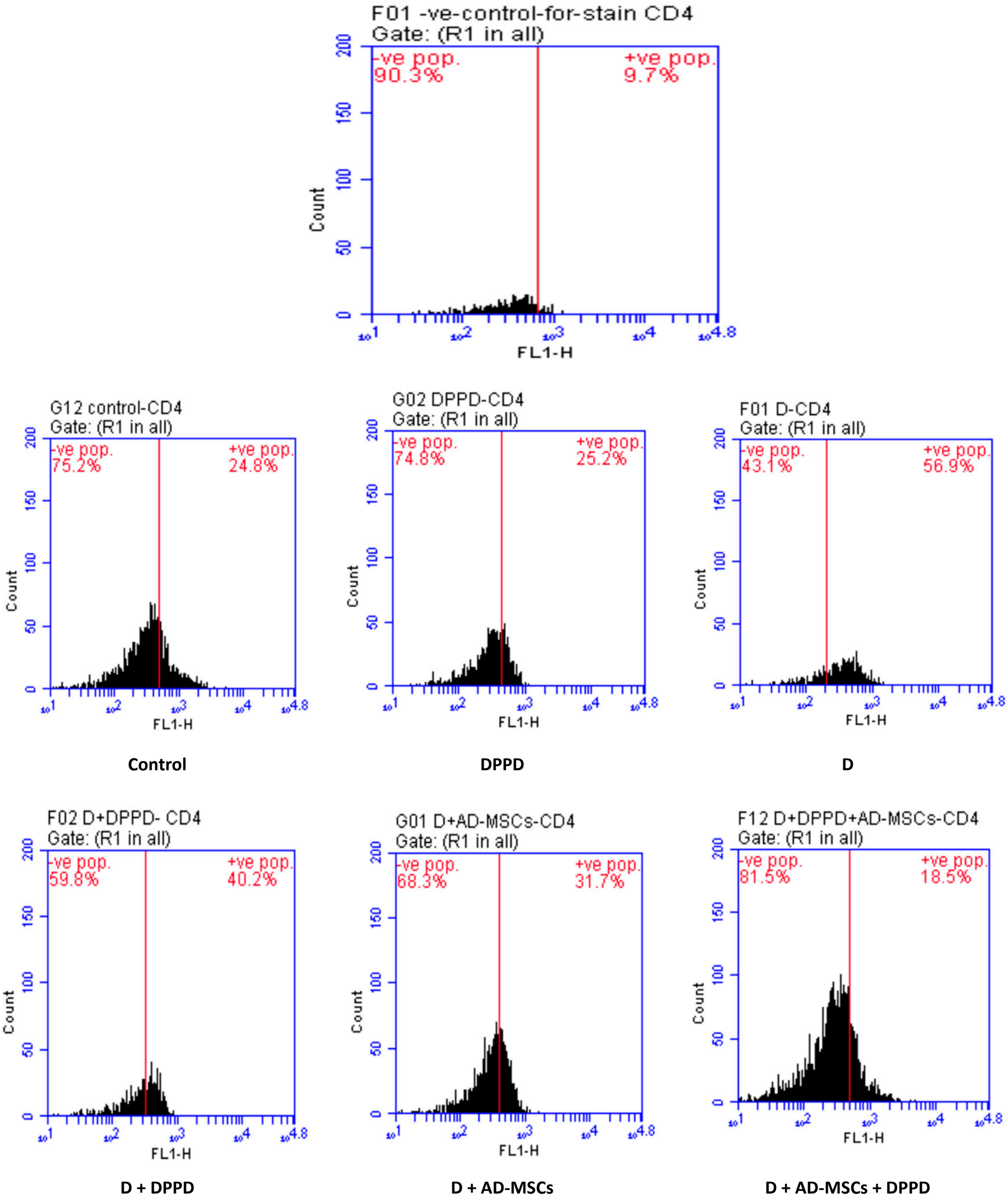

Figure S2a. Pancreatic CD4+ %.

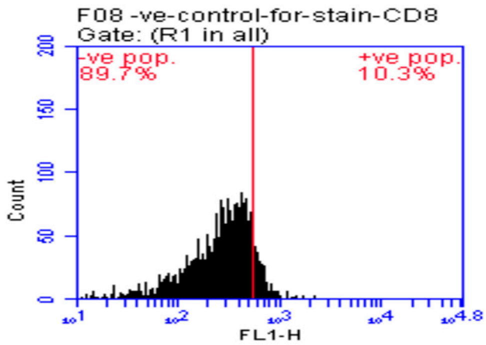

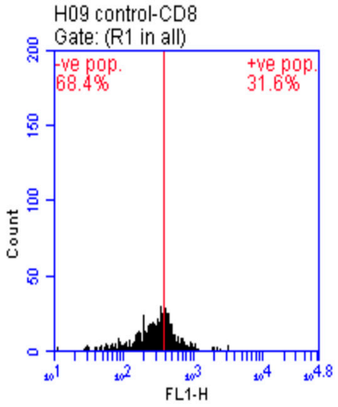

control

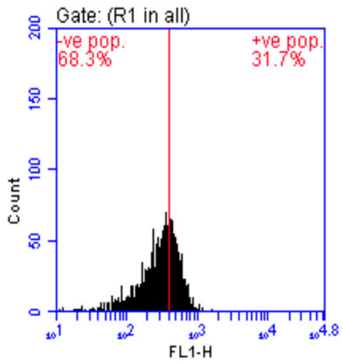

DPPD

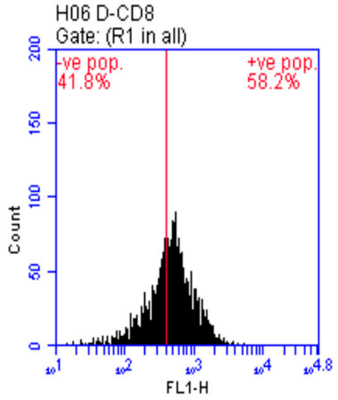

D

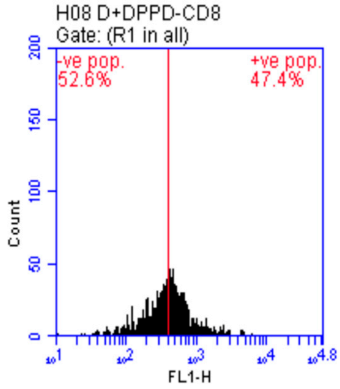

D + DPPD

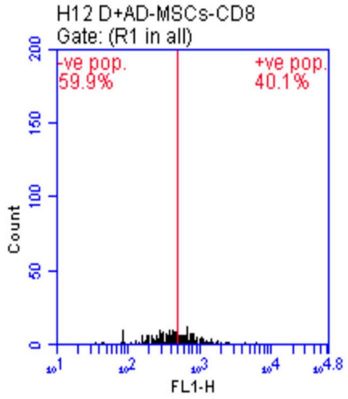

D + AD-MSCs

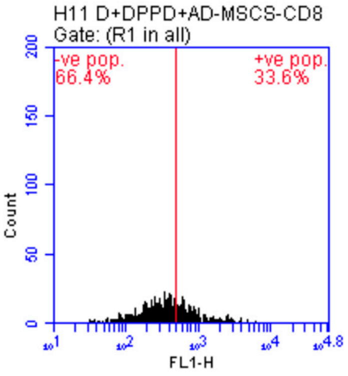

D + AD-MSCs + DPPD

Figure S2b. Pancreatic CD8+ %.

Figure S2 (a-b). Pancreatic T-Helper (CD 4^{+}) and T-Cytotoxic (CD 8^{+}) %.

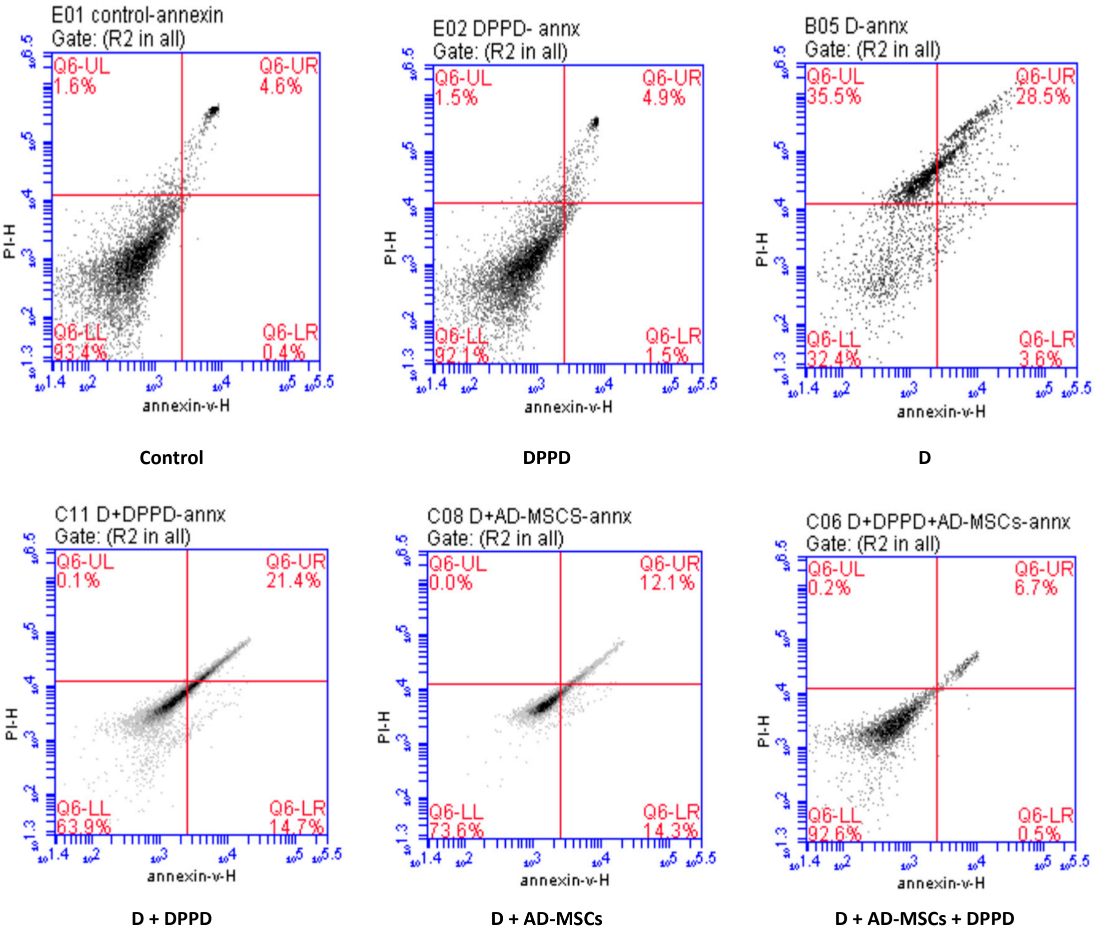

Figure S3a. Pancreatic annexin V %.

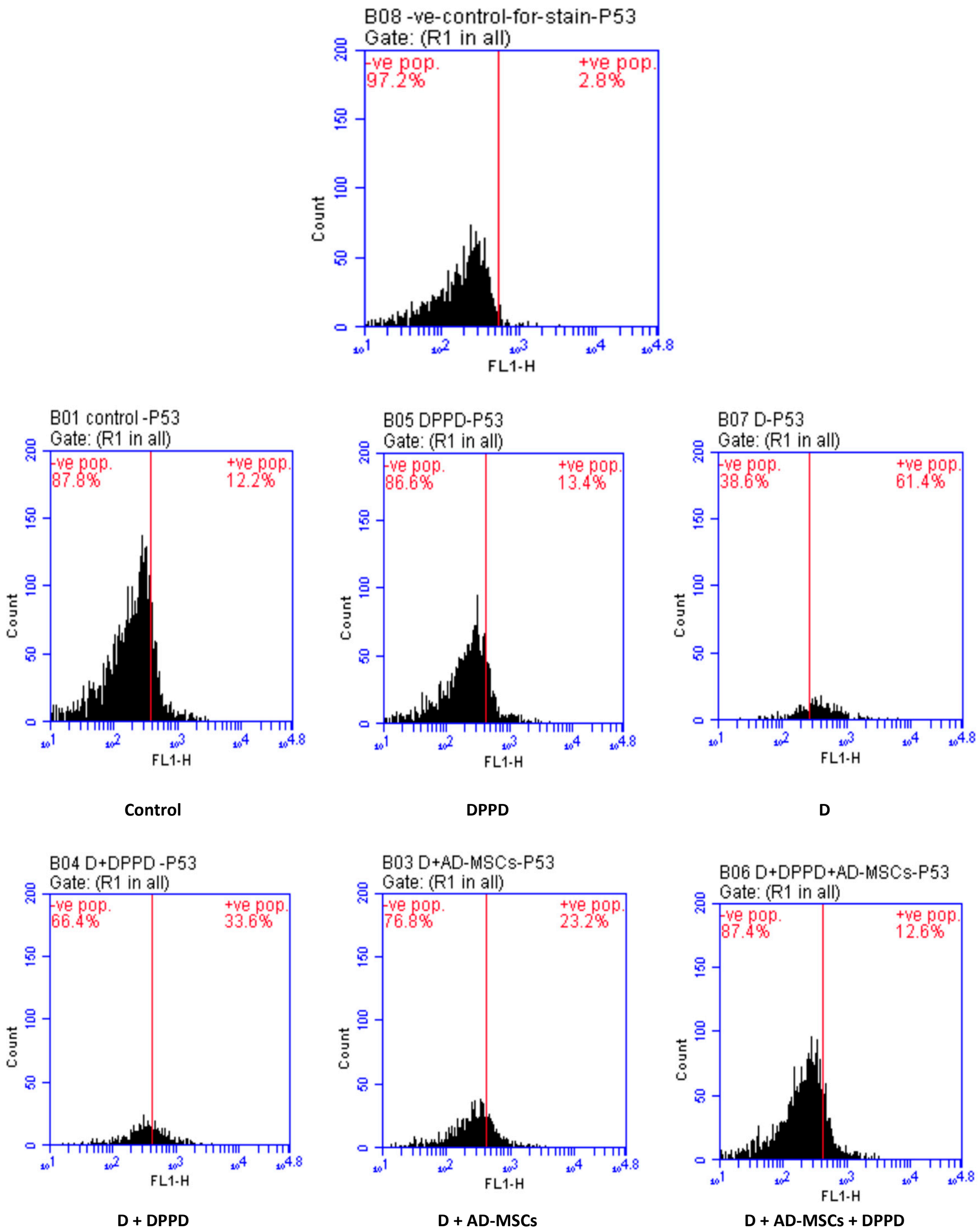

Figure S3b. Pancreatic P53 %

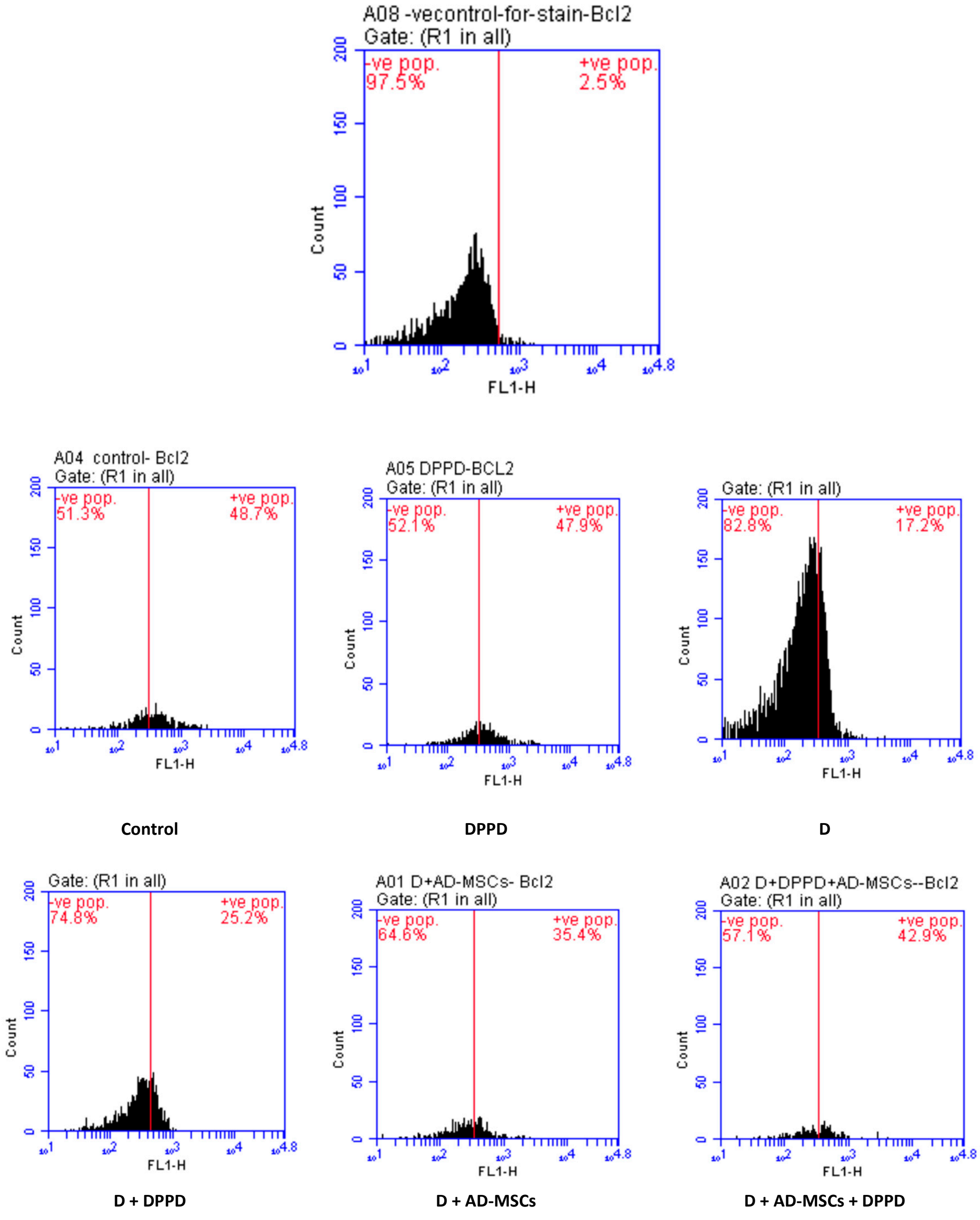

Figure S3c. Pancreatic Bcl-2 %.

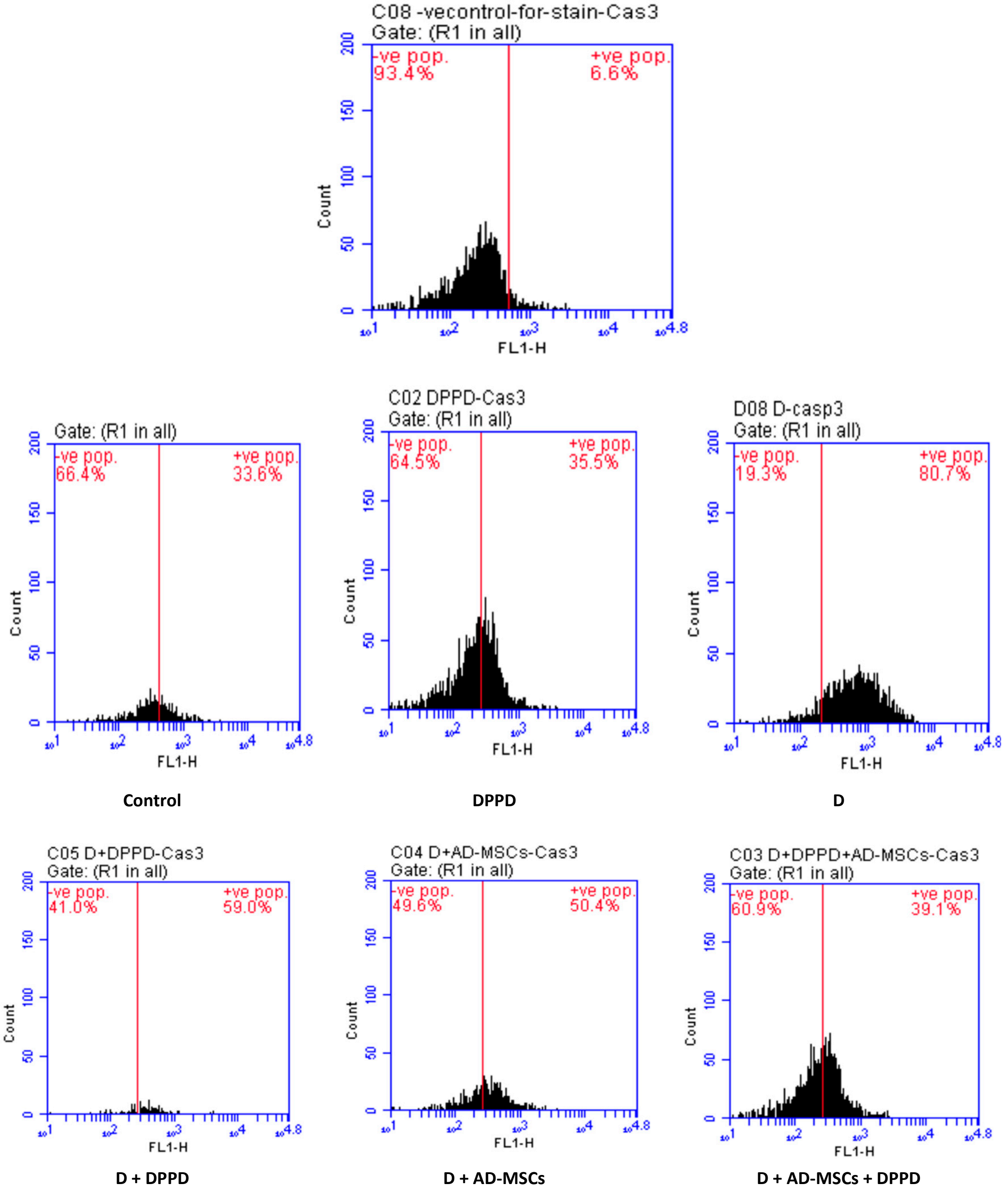

Figure S3d. Pancreatic caspase 3 %.

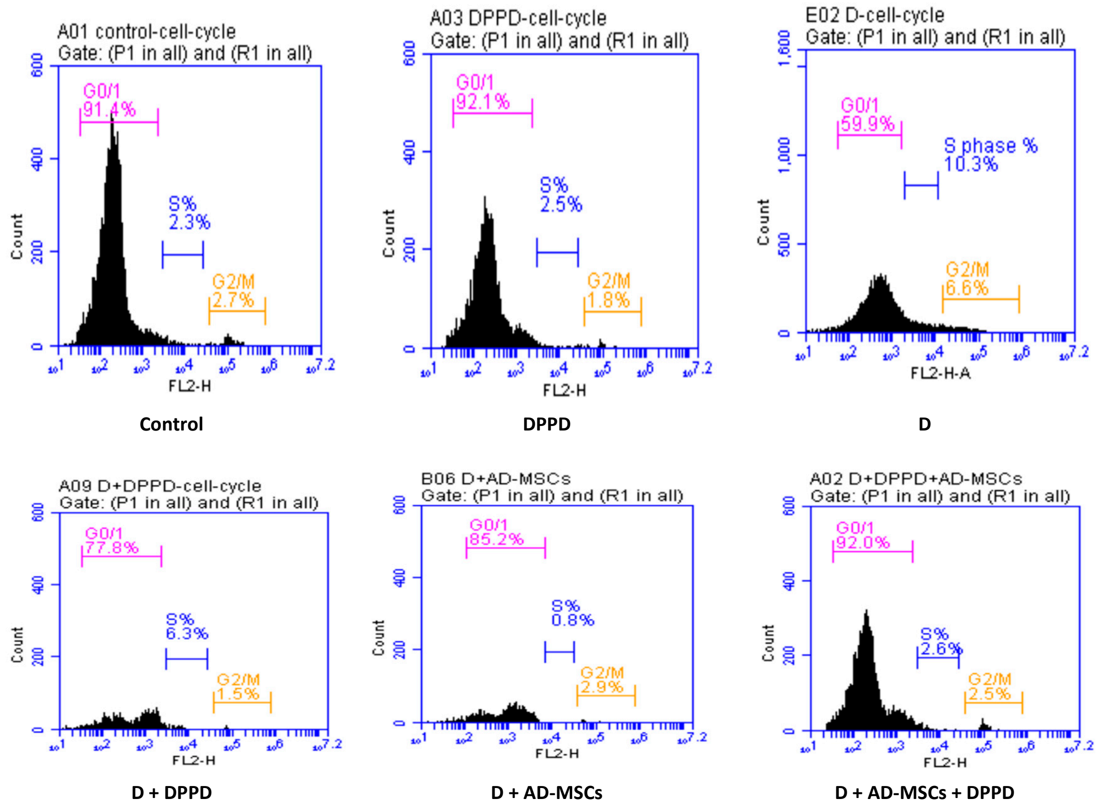

Figure S3e. Pancreatic viable cells (G0/G1) %.

Figure S3 (a-e). Pancreatic annexin V, P53, caspase 3, Bcl-2 & viable cells (G0/G1) %.

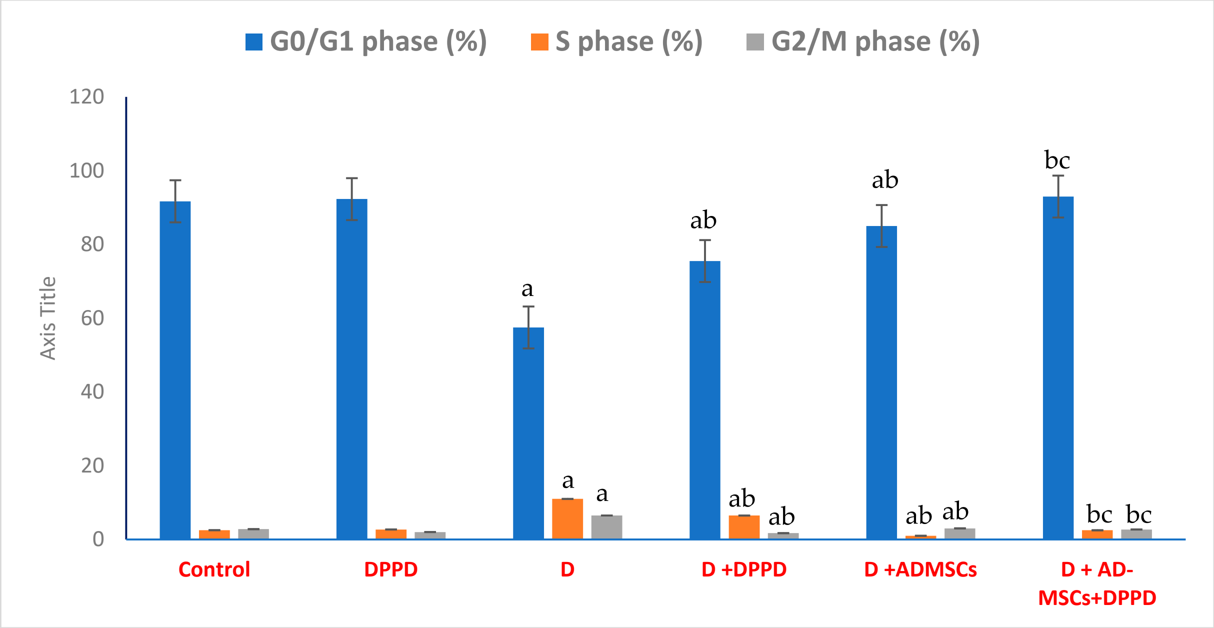

Figure S4. Pancreatic cell cycle %. Values expressed as ± SEM (n = 6). [a,b] & [c] are Significant differences (P ≤ 0.05) compared to control, diabetic untreated and DPPD-diabetic treated groups, respectively. % is the percent of change compared to the diabetic group.

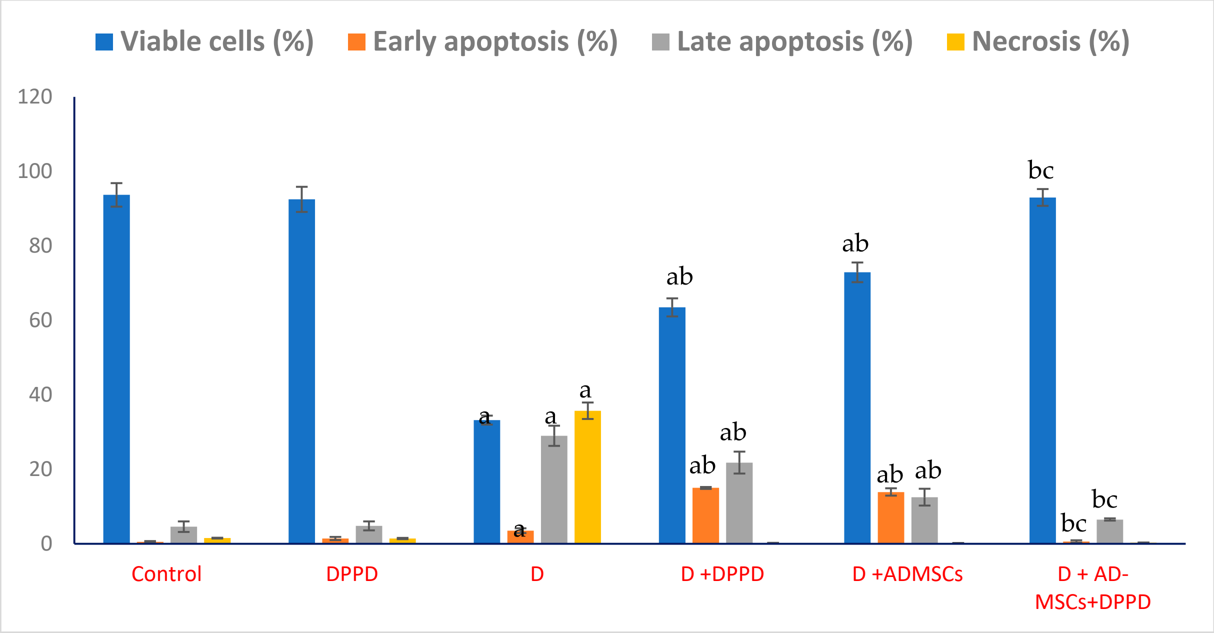

Figure S5. Pancreatic annexin V %. Values expressed as ± SEM (n = 6). [a,b] & [c] are Significant differences (P ≤ 0.05) compared to control, diabetic untreated and DPPD-diabetic treated groups, respectively. % is the percent of change compared to the diabetic group.
